# Identifying elemental genomic track types and representing them uniformly

**DOI:** 10.1186/1471-2105-12-494

**Published:** 2011-12-30

**Authors:** Sveinung Gundersen, Matúš Kalaš, Osman Abul, Arnoldo Frigessi, Eivind Hovig, Geir Kjetil Sandve

**Affiliations:** 1 Department of Tumor Biology, The Norwegian Radium Hospital, Oslo University Hospital, Montebello, 0310 Oslo, Norway; 2 Computational Biology Unit, Uni Computing, Thormøhlensgate 55, 5008 Bergen, Norway; 3 Department of Informatics, University of Bergen, Thormøhlensgate 55, 5008 Bergen, Norway; 4 TOBB University of Economics and Technology, Ankara, Turkey; 5 Statistics For Innovation, Norwegian Computing Center, 0314 Oslo, Norway; 6 Department of Biostatistics, Institute of Basic Medical Sciences, University of Oslo, Blindern, 0317 Oslo, Norway; 7 Institute for Medical Informatics, The Norwegian Radium Hospital, Oslo University Hospital, Montebello, 0310 Oslo, Norway; 8 Department of Informatics, University of Oslo, Blindern, 0316 Oslo, Norway

## Abstract

**Background:**

With the recent advances and availability of various high-throughput sequencing technologies, data on many molecular aspects, such as gene regulation, chromatin dynamics, and the three-dimensional organization of DNA, are rapidly being generated in an increasing number of laboratories. The variation in biological context, and the increasingly dispersed mode of data generation, imply a need for precise, interoperable and flexible representations of genomic features through formats that are easy to parse. A host of alternative formats are currently available and in use, complicating analysis and tool development. The issue of whether and how the multitude of formats reflects varying underlying characteristics of data has to our knowledge not previously been systematically treated.

**Results:**

We here identify intrinsic distinctions between genomic features, and argue that the distinctions imply that a certain variation in the representation of features as genomic tracks is warranted. Four core informational properties of tracks are discussed: gaps, lengths, values and interconnections. From this we delineate fifteen generic track types. Based on the track type distinctions, we characterize major existing representational formats and find that the track types are not adequately supported by any single format. We also find, in contrast to the XML formats, that none of the existing tabular formats are conveniently extendable to support all track types. We thus propose two unified formats for track data, an improved XML format, BioXSD 1.1, and a new tabular format, GTrack 1.0.

**Conclusions:**

The defined track types are shown to capture relevant distinctions between genomic annotation tracks, resulting in varying representational needs and analysis possibilities. The proposed formats, GTrack 1.0 and BioXSD 1.1, cater to the identified track distinctions and emphasize preciseness, flexibility and parsing convenience.

## Background

Recent ChIP and high-throughput sequencing technologies are currently generating functional annotations at unprecedented speed and resolution. The availability of detailed protein binding locations, DNA methylation, histone modifications, DNA variations of individuals, and more for different tissues and conditions, provides the basis for a plethora of representational formats of genome wide data. Adding to this, new technologies for assessing the three-dimensional structure of the DNA, such as Hi-C [1], introduce the concepts of distance measures between different parts of a genome, opening up a whole new set of representational complexity.

Several efforts have been attempted at defining general formats for the textual representation of genome annotation data. One such format is the General Feature Format (GFF), currently in version 3 [2]. Other generic formats are provided in connection to the UCSC Genome Browser [3], the Browser Extensible Data format (BED), bedGraph and WIG, among others. One reason for the different formats is that different properties are required, often in order to support information related to specific domains, technologies or experimental methods. Consider for instance the BED15 format by UCSC. This is an extension of the BED format, adding 3 columns in order to represent microarray expression data [4]. Other examples are the Gene Transfer Format (GTF) [5] for gene tracks and the Genome Variation Format (GVF) [6] for DNA variant files, both based on the GFF format.

Another reason behind the proliferation of formats seems to be an issue of practicality. Certain types of genome annotations, or genomic tracks, are more efficiently and elegantly represented by certain data formats. Consider a track of DNA melting temperatures, *i.e*. an algorithmic prediction of the denaturation temperature for each base pair of the genome, *e.g*. [7]. Representing such a track in the Wiggle format (WIG) would take around 20 GB for the human genome. The exact same information could be represented in the bedGraph format, but the file size would then expand to around 100 GB. In this case, the file would contain much redundant information, such as repeated chromosome declarations, and start and end positions that are always increased by one for each line. The help pages at the UCSC Genome Browser explicitly recommend the WIG format for "dense, continuous data" and bedGraph for "continuous data that is sparse or contains elements of varying size" [8]. From this it seems that, at an abstract level, there may exist fundamental distinctions between track data, such that warrants the use of particular textual formats. We are, however, not aware of any systematic discussion of such distinctions in the literature.

Expanding on this notion of systematic distinctions between track data, it seems that such distinctions also warrant differences in which analyses are applicable. It is for instance meaningful to ask whether SNPs fall inside exons, but it is not meaningful to ask whether SNPs fall inside melting temperature. Conversely, one can ask whether SNP locations have high melting temperatures, but not whether SNPs have high exons. This indicates that there may be some form of abstract grammar, where each track defines a set of informational properties, and each analysis only makes sense on certain sets of informational properties for the tracks in question.

In this paper, we start with a clarification of basic nomenclature. We then discuss how the presence of different core informational properties of a track can be used to delineate fifteen different types of tracks at an abstract level. The fifteen track types encompass most existing data formats, in addition to open up for data sets making use of cross-positional linking, *e.g*. data sets based on the three-dimensional structure of DNA. We continue by reviewing common, generic formats, in tabular, XML-based, or binary form, and discuss how they fit with the proposed track types. This is followed up with the proposal of a new tabular format and an updated XML format for track data. These formats build closely on previous ones, but obey the distinctions between types of tracks. Finally, we discuss supporting tools for the proposed formats, including a code base supporting the storage of tracks in efficient binary format, illustrating how the formats can be pragmatically applied in high-speed analyses.

## Results and Discussion

### Definitions

A reference genome may be abstracted as a line-based coordinate system. To build on this powerful metaphor, we use the term *genomic track *(or, in short, *track*, as used by the UCSC Genome Browser [3]) to refer to a series of data units positioned on such a line. The basic informational unit is called a *track element*, that is, a unit of data with associated genomic coordinates that may or may not be explicitly specified. A track element is to be thought of as a mathematical or implementational abstraction, in tabular formats typically represented as a single data line. Although the concept of genomic tracks is most useful for describing data that refer to a single reference genome, the meaning carries easily over to datasets referring to multiple reference genomes, or to contigs or scaffolds of partially assembled genomes.

We further define a *genome feature *as a track element or set of track elements comprising a biological unit, *e.g*. a specific gene, of a certain feature type, *e.g*. genes. The term *biological unit *is to be understood broadly and should also include experimental results, algorithmic predictions and similar concepts, such as defined under *sequence feature *in the Sequence Ontology [9]. Note that a feature, *e.g *a gene, may be composed of several track elements, *e.g*. representing the exons of that gene. Often, a complete genome annotation, *i.e*. features of many feature types connected to a genome, are collected into a single file. This complicates the comparison of different feature types, creating the need for filtering such a file for the appropriate feature types prior to analysis. On the other hand, restricting a track to contain only a single feature type may reduce the information. For example, the connection between genes and their exons is lost if the two feature types are stored as separate tracks. We thus define a *genomic track *more specifically as set of track elements of one or several feature types, defined over an appropriate genome-scale coordinate system, where the set of feature types constitutes a pragmatic unit for analysis. A genomic track is then, in our view, defined in relation to an analytical purpose, whether explicitly defined or only suggested; this, in contrast to a data file used mainly for storage, which should be considered more as a flat file database.

### Core informational properties of tracks

A genomic track consists of a set of track elements and, for each element, describes a set of properties, such as an identifier, a quality score or the method used. The positional information of a track element is obligatory for any genomic track and can be interpreted generically across tracks. The position of a track element is often encoded as a pair of start and end coordinates. However, when looking at genomic tracks from the perspective of information content, we find it fruitful to identify the positional information equivalently as the *lengths *of the track elements and the *gaps *between them, both measured in base pairs. As the positional information is essential and generic, we refer to gaps and lengths as *core informational properties *of the track.

A genomic track may also carry a main value associated with each track element, for instance the measured expression of a gene or the copy number of a genomic region. We thus include *values *among the core informational properties. This main value can be a number (*e.g*. the expression of a gene), a binary value (*e.g*. if the element is considered case or control), a category (*e.g*. the feature type), a character (*e.g*. the allele variant of a SNP), or a list of values (*e.g*. gene expression for a set of patients).

Lastly, a track element may be connected to other track elements located at different locations on the genome. This is critical for three-dimensional tracks, as locations that seem far apart when the DNA is unwound, could still be co-located in the nucleus. The corresponding core informational property of a track is then *interconnections*. The interconnections, or edges, are either directed or undirected, possibly with an attached weight value.

### Fifteen genomic track types

All four core informational properties (*gaps*, *lengths*, *values*, and *interconnections*) will not always be defined for a track. Consider, for instance, a track of viral insertion points on a genome. As it makes no sense to talk about the length of an insertion point, such a track will not have the lengths property defined. Similarly, a track of single nucleotide polymorphisms (SNPs) will only contain elements that refer to single discrete positions on the genome. The track elements will, however, have associated values denoting the respective alleles. Consider also the DNA melting map, a track where a temperature value is assigned to every base pair of the genome [7]. As temperature values, *i.e*. track elements, are defined for every consecutive position of the genome, there is never any gaps between the elements. Also, the elements refer to single base pairs and have no lengths. Thus, a track of DNA melting will have neither the *lengths *nor the *gaps *property defined, only the *values *property (denoting temperature).

Four core properties, being defined or not, gives 2^4 ^= 16 distinct combinations. Assuming that a genomic track always consists of track elements with the same core properties, we can distinguish tracks on the basis of which combination of core properties are defined. For one of the sixteen combinations, no core properties are defined. It is thus of no interest, hence reducing the set to fifteen combinations.

**Figure 1:**
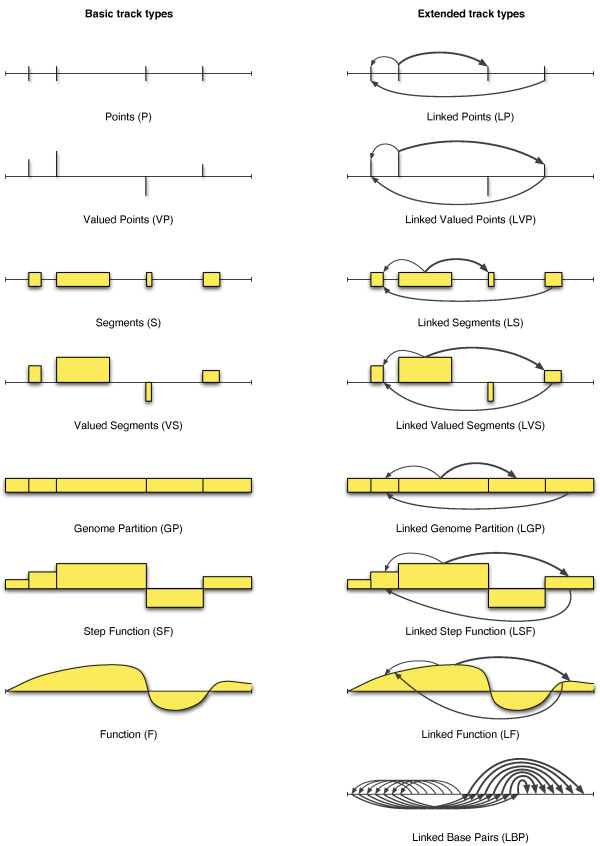
**Illustration of the geometric properties of the fifteen track types**. The base line is a genome, or a sequence, on which the tracks are defined. Vertical lines represents positions, while horizontal lines represent the lengths of the track elements. Gaps are thus illustrated by any empty areas between the elements. Values are represented by the height of the vertical lines. Interconnections are represented by arrows, the thickness of which correspond to the weight of the edge.

Looking closely at the fifteen combinations, an interesting pattern appears. Figure 1 shows an illustration of the informational contents of each combination. As every combination denotes a particular geometric configuration, strikingly distinct from the others, we refer to tracks of the different combinations as having different *track types*. The concept of dividing genomic tracks into track types was partially introduced in [10], but has now been expanded from five to fifteen track types.

Looking at the top left of Figure 1 and going downward, we start at the base case where the only core informational property is the gaps between the track elements. In this case, each track element represents an exact base pair location on the genome, denoting *e.g*. viral insertion sites. We call this track type *Points (P)*. Adding informative values to this case, *e.g*. associating SNPs with allele frequencies, we get the track type *Valued Points (VP)*. In the next two cases, the lengths property is added, resulting in the track types *Segments (S) *and *Valued Segments (VS)*. Segments are probably the most common track type of existing tracks, representing common features such as genes or exons. Valued segments could, for instance, denote genes with associated expression levels.

Moving on, we remove the *values *and *gaps *properties, leaving only *lengths*. Such tracks consist of segments covering all base pairs of the genome, *i.e*. a partition of the genome into potentially unequal pieces. Hence, the track type is called *Genome Partition (GP)*. Basic examples of this track type are the partition of a genome into chromosomes or cytobands. Adding a value to each part of a partition creates a *Step Function (SF)*, covering the whole genome with values. Basic examples of such tracks are tracks denoting results of tiling microarrays, providing that any gaps or overlaps between the tiles are ignored. Removing the *lengths *core property, the step function track is transformed into a track of type *Function (F)*, where every base pair has an associated value. Examples of function tracks are tracks with close dependency on the genome sequence, such as GC content tracks, or predictions of melting temperatures, as outlined above. We call the seven track types outlined here for *basic track types*.

The fourth core informational property, *interconnections*, can be envisioned as an orthogonal extension to the previous discussion. Adding interconnections, or edges, to the seven track types previously outlined (first column in Figure 1) defines linked versions of the same track types, *e.g*. *Linked Segments (LS) *or *Linked Step Function (LSF) *(second column of Figure 1). Although tracks that include interconnections are presently in little use, enough datasets exist to warrant the definition of all the linked track types, at least for completeness. For example, the recent Hi-C dataset of Dekker et al. [4] partitions the genome into 1 Mbp regions (for the genome-wide case), where each pair of regions has an associated proximity value. This dataset is then of type *Linked Genome Partition (LGP)*, where every region has a weighted edge to all other regions. More traditionally, one could envision a gene/protein pathway being represented as gene segments, perhaps also with associated expression data, being linked together with directed edges representing associations (binding, activation, inhibition, etc.). This would be of type *Linked Valued Segments (LVS)*. Note that a track type is considered linked if *at least some *track elements are interconnected.

**Figure 2:**
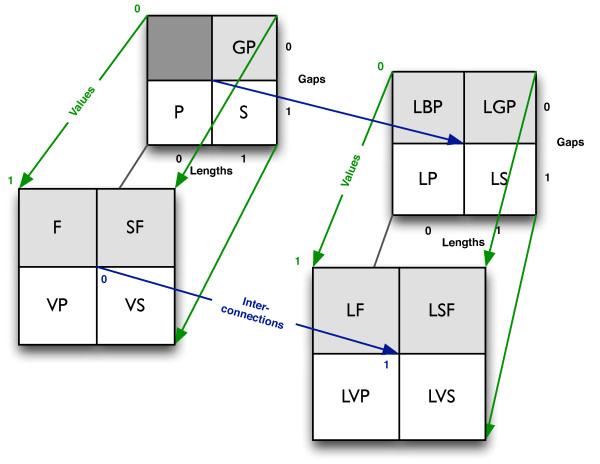
**Four-dimensional matrix mapping the relations of the fifteen track types**. Each dimension represents the exclusion (0) or inclusion (1) of one of the four core informational properties: gaps, lengths, values and interconnections. The track type abbreviations in the top-left box are: *Genome Partition (GP)*, *Points (P) *and *Segments (S)*; in the bottom-left box: *Function (F)*, *Step Function (SF)*, *Valued Points (VP) *and *Valued Segments (VS)*; in the top-right box: *Linked Base Pairs (LBP)*, *Linked Genome Partition (LGP)*, *Linked Points (LP) *and *Linked Segments (LS)*; and in the bottom-right box: *Linked Function (LF)*, *Linked Step Function (LSF)*, *Linked Valued Points (LVP) *and *Linked Valued Segments (LVS)*. The track types with white background (with gaps) are the *sparse *track types, while the ones with grey background (without gaps) are the *dense *track types. See Figure 1 for a geometric illustration of the track types.

To complete the picture, a last track type needs to be defined. If only the *interconnections *core property is defined, track elements do not have gaps between them, lengths, or values. All base pairs are then track elements, with each base pair connected to other base pairs by edges, hence the name *Linked Base Pairs (LBP)*. Thinking in term of graphs, all base pairs will thus be nodes, although not all nodes need to have any edges. This, in contrast to the track type *linked points*, which limits the nodes to a specified set of points. The track type *Linked Function (LF) *is similar to *linked base pairs*, only adding an associated value to each base pair (node). The *linked base pairs *track type is mostly suggestive at this point, but at least theoretically, this would be the track type of the perfect three-dimensional track, mapping the distance between all base pairs of a genome. Another example of a track of this kind is the representation of a randomization of a genome, with each edge representing the positional relocation of a base pair. We refer to the eight linked track types as the *extended track types*. Figure 2 shows an overview of the relations between the fifteen track types and the combination of core informational properties defined.

### Formal model of genomic tracks

Formally, we base the discussion of track types on a specific mathematical model of genomic tracks. We treat the genomic coordinates as forming a discrete metric space on the natural numbers, defined by the discrete metric d:

(1) d(a,b)=∣a-b∣+1,a, b∈ℕ

The genomic coordinates in the model are thus isolated points. A segment or interval starting at a position *a *and ending at *b *is defined as the subset *S *of natural numbers where:

(2) S(a,b)={s∈S∣a≤s≤b∧b>a}

The length of a segment is defined by the metric *d*, and is equal to the number of elements in the set. The length of the segment *S*(1, 3) = {1, 2, 3} is thus *d*(1, 3) = |1 - 3| + 1 = 3 = |*S*(1, 3)|. Transferred to the biological domain, the length of a segment is the number of base pairs covered by the segment. The end position of a segment must be larger than the start position. We thus exclude segments of length 1 from the model, as such segments would be exactly equal to a point, e.g. the set of a single number:

(3) P(a)={p∈P∣p=a}

From the set notation follows that a point *P *can be precisely defined as falling inside a segment *S *if and only if *P *⊂ *S*. Two segments, on the other hand, may partially overlap. A function is precisely defined as a mathematical function from genomic coordinates to corresponding values, *e.g*. *f *= ℕ → ℝ. A step function is similarly a function from disjoint intervals covering the entire domain to corresponding values.

### Analysis dependency on track types

As each of the fifteen track types implies a set of core informational properties, a track type also poses a limit to which analyses are appropriate for a track. It makes sense to calculate the base pair coverage of a track of genes (type: *segments*), but not for a track of SNPs (type: *valued points*), which should instead be counted. This logic also carries on to analyses applied to more than one track. Consider, for the sake of simplicity, only five of the fifteen track types. If we select two tracks, each of one of these five types, we get 15 combinations, provided that the order of the tracks is not important. Each of these combinations could then define a set of appropriate analyses. Table 1 provides analysis examples for many of the pairwise combinations of the five track types *points*, *segments*, *function*, *valued points*, and *valued segments*. Although assigned to a single combination of track types, an analysis may often be meaningful for a set of such combinations. For instance, asking whether the points of one track are located inside the borders of the segments of another track (*points *vs *segments*) will trivially also give meaning where one or both of the tracks has associated values (*e.g*. *valued points *vs *valued segments*). Also, it could give meaning to ask whether small segments of one track are located inside the borders of the segments of another track (*e.g*. for the *segments *vs *segments *combination). The correspondence between the track types and possible analyses are at the core of the idea of track types. Although storing data sets as efficiently as possible is an important aspect, the bioinformatics field is currently lagging more in terms of general understanding and standards for analyzing data sets in meaningful ways. It is our hope that the definition of track types will help in this regard.

**Table 1:** Relation between analyses and track types

	Points	Segments	Function	Valued Points	Valued Segments
**Points**	Different frequencies?	Located inside?	Higher values at locations?		Located in highly valued segments?
	**Segments**	Overlap?	Higher values inside?		
		**Function**	Correlated?		
			**Valued Points**	Nearby values similar?	Categories differentially located in targets?
				**Valued Segments**	

Examples of analyses for different combinations of track types (using only five of the fifteen defined track types). Note that many of these analyses are valid for several (though not all) combinations, and are assigned to what we consider the most typical combination for the analysis. All these analyses are carefully described significance tests [10], available online at the Genomic HyperBrowser [28]

### Existing representational formats

Existing formats for representing genomic tracks can broadly be divided into three groups: textual formats, binary formats, and XML formats. Often textual and binary formats are closely connected, such as the SAM and BAM formats for read alignments [11]. This duality is due to the different advantages of the two forms. Textual formats are often humanly readable and simpler to parse and manipulate than their binary alternatives. The binary formats, on the other hand, are more compact and more efficient to use, often incorporating indexing schemes for fast random access to data. XML formats aim to bridge this gap by defining data structures that can exist in both textual and binary forms. Note that we limit the discussion to formats that aim at being general, in one form or another, thus excluding formats that are special to a particular technology or platform.

**Figure 3:**
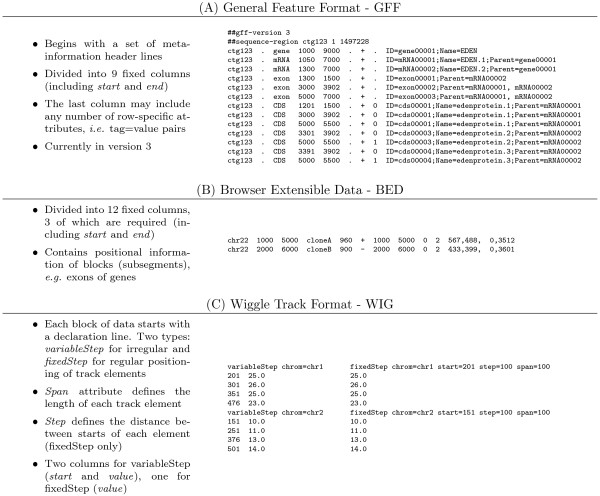
**Overview of three common tabular formats**. A) Generic Feature Format (GFF). The example file is a reduced version of the main example of the GFF version 3 specification [2]. B) Browser Extensible Data format (BED). The example file is fetched from the specification of the format at UCSC [4]. C) Wiggle Track Format (WIG) [8]. The example files show the two subformats *variableStep *and *fixedStep*. The track elements in the variableStep file covers single base pairs (span = 1, as default) and contains *sparse *data. For the fixedStep file, the step attribute is equal to the span attribute. The fixedStep file thus contains *dense *data. Figure 4 shows GTrack conversions of these example files.

The large majority of formats for genomic data are textual, and the large majority of the textual data formats are tabular, that is, they consist of tab-separated columns. Three of the most common tabular formats are Generic Feature Format (GFF) [2], Browser Extensible Data format (BED) [4] and Wiggle Track Format (WIG) [8]. Figure 3 shows an overview of these three tabular formats, with example files.

A main reason for the popularity of tabular formats is that they are inherently simple to create and read, both manually and by computers. This has been a major asset in the field of bioinformatics because of the widespread use of both *ad hoc *scripting and WYSIWYG editing in spreadsheet software (such as Microsoft Excel). Still, the abundance of different formats, together with the increased complexity of particular formats, creates practical problems when *e.g*. creating new tools.

XML formats represent a way of letting go of the entire process of custom and explicit parsing of files. In particular when an XML format is specified by a dedicated XML Schema (abbreviated XSD, from XML Schema Definition), the data included in an XML document can be automatically transformed into convenient runtime data objects. XML formats are much used in connection with Web services, XML databases, or serializations of object models, but there have so far been only a few XML formats used for exchanging sequence-feature data. The Distributed Annotation System [12] uses the DASGFF XML format, which is similar to the tabular GFF. Web services for feature prediction at CBS [13] have been using a common XSD-based output format that has been inspired by GFF. Numerous Web services and databases define their own XML formats for annotation data, such as the UniProt XML [14] or the ELMdb Web service [15]. BioXSD version 1.0 has defined a format for sequence features that is expressive enough to be able to substitute the majority of other feature formats [16]. The main disadvantages of using XML for genome-scale annotations have been the verbosity of the textual serialization of XML data and the large memory usage of most of the libraries parsing XML. The recent W3C standard for highly optimized binary representation of XML - the Efficient XML Interchange (EXI) format [17] - promises to solve these problems.

Binary formats are often used internally in software systems, and not necessarily provided as public formats. Some exceptions to this are the aforementioned BAM, as well as the bigBed and bigWig formats [18]. The last two formats are binary versions of the BED and WIG format, respectively, providing efficient storage and indexing capabilities, allowing users to store large tracks on their own computers, while a server requests only the parts needed for analysis or visualization. Another binary format is the USeq Compressed Binary format [19] focusing on tight compression of tabular data files of different types, while keeping them in an indexed structure.

As Figure 3 illustrates, different formats support different combinations of the core informational properties, and hence, different track types. Table 2 provides an overview of which of the basic track types are covered by some common formats. As each of the different groups of formats (tabular, XML, and binary) has advantages in distinct scenarios and communities, one would ideally like to select three formats that cover all track types, one from each group. Unfortunately, no common formats do. One option would be to extend an existing format to support all track types. A main reason for such an extension would be to be able to make use of the plethora of tools and parsers already available. In the case of XML formats, the existing BioXSD 1.0 format was found to be easily extensible to support all track types. In the case of tabular formats, however, the only major format to support extensions is GFF, through the attribute column. However, using GFF to represent *e.g*. tracks of type *function *would be highly impractical. Each base pair would then be represented by a data line of nine columns, wasting considerable amounts of space. The remaining option is then to create a new tabular format. In order for the introduction of a new format to be justified, such a format should have the potential to replace at least some of the existing formats, in addition to having the extensibility required to meet future needs when new types of data appear. As binary formats are often not independent formats, but typically linked to tabular ones, we will not focus on such formats here. We thus present a pair of general formats aware of all track types, one of which is tabular and the other based on XML. The tabular format, GTrack 1.0, is a new format that builds closely on the BED and WIG formats, while adding support for extensions in a similar fashion as in GFF. The XML format is a successor of the existing BioXSD 1.0 format. Besides catering to a broader user base, presenting "track type"-compliant formats of both kinds illustrates that the fundamental concepts of track type are independent of implementation. The primary goals for the formats are to support all track types systematically, to allow custom extensions, and to provide efficient storage, while at the same time focusing on simple parsing and manipulation of files.

**Table 2:** The track types supported by existing tabular, binary and XML formats

Format	**Ref**.	Data	**Repr**.	P	S	VP	VS	GP	SF	F	L	Strand	#Cols	Value type
GFF3/GTF	[2]	General	Tab.	✓^1^	✓	✓^1^	✓				^2^	✓	9	Float^3^
BED/bigBed	[4]	General	Tab./Bin.	✓^1^	✓	✓^1^	✓				^2^	✓	3-12	Int(0-1000)/string^4^
BED15	[4]	Microarray	Tab.			✓^1^	✓				^2^	✓	15	List of floats^5^
bedGraph	[4]	General	Tab.			✓^1^	✓						4	Float
WIG/bigWig (fixedStep)	[8]	General	Tab./Bin.			✓	✓		✓	✓			1	Float
WIG/bigWig (variableStep)	[8]	General	Tab./Bin.			✓	✓						2	Float
CNT	[36]	Copy number	Tab.			✓							4	Float
Personal Genome SNP	[4]	Variation	Tab.			✓^1^	✓						7	String^6^
VCF	[37]	Variation	Tab.			✓	✓						≥ 8	String^6,3^
GVF	[6]	General/Variation	Tab.	✓^1^	✓	✓^1^	✓				^2^	✓	9	Float^3^
PSL	[4]	Alignment	Tab.		✓		✓					✓	21	Int^7^
SAM/BAM	[38]	Alignment	Tab./Bin.		✓		✓					✓	11	Int/string^8^
BioHDF	[39]	Alignment	Bin.		✓		✓					✓	11	Int/string^8^
MAF	[4]	Multiple Alignment	Tab.		✓		✓				^9^	✓	2-7	Float/string^8^
FASTA	[40]	Sequence	Text							✓			N/A	Char
DAS XML	[12]	General	XML	✓^1^	✓	✓^1^	✓				^2^	✓	N/A	Float
BioXSD 1.0	[16]	General	XML	✓^10^	✓^10^	✓^10^	✓^10^				✓^11^	✓	N/A	Float^12^
USeq	[19]	General	Bin.	✓	✓	✓	✓					✓	N/A	Int/float/string
Genomedata	[41]	General	Bin.			✓	✓		✓	✓			N/A	Int/float/char

The track type abbreviations are as follows: *Points (P)*, *Segments (S)*, *Valued Points (VP)*, *Valued Segments (VS)*, *Genome Partition (GP)*, *Step Function (SF)*, and *Function (F)*. *L *refers to any of the linked track types. The table also denotes whether the format supports specification of strand, the number of columns of the tabular formats, and the type of the dominant value, if any.

^1 ^Points are specified using both start and end values. There is no way of specifying that a file contains only points.

^2 ^Only a special case of linked segments is supported, namely part-of relationships, such as en exon being a part of a gene.

^3 ^The chosen value type refers to what may be considered the main score column of the format. The format also includes a configurable column containing values that may be extracted by specialized parsers.

^4 ^We limit the bigBed format to the standard BED columns for simplicity, as the bigBed format is highly customizable through the use of AutoSQL configurations.

^5 ^The float values represent a set of gene expression values from microarray experiments.

^6 ^The values represent the possible alleles at a SNP position. Also, the allele frequencies and quality scores are reported and could be used as values.

^7 ^*E.g*. the number of bases that match/do not match.

^8 ^*E.g*. the mapping quality or the aligned sequence itself.

^9 ^Links to alignments in other genomes.

^10 ^There is no way of specifying that a record contains only points or only segments.

^11 ^No *weights *are supported in BioXSD 1.0.

^12 ^Numerical values are always signed double precision floats (8 bytes). A limited set of other value types is also allowed (*e.g*. sequence variation and alignments).

### GTrack: Type-aware tabular format

We here introduce a new tabular track format: the GTrack format, short for both "Genomic Track" and "Generic Track". The GTrack format supports all fifteen previously defined track types, illustrated in Figures 1 and 2. A GTrack file includes a *column specification line*, specifying the names of all the columns in the file. Each track type has a one-to-one correspondence to a combination of core columns being present in the column specification line, as detailed in Table 3. The four core informational properties are represented by the four core reserved columns in such a way that the existence of each core column (*start*, *end*, *value*, and *edges*) corresponds to a core property being defined (*gaps*, *lengths*, *values*, and *interconnections*, respectively):

**Table 3:** Overview of the reserved columns in the GTrack format and their associations to track type

Core property:			Gaps	Lengths	Values			**Interc**.
**GTrack column(s):**	**genome**	**seqid**	**start**	**end^1^**	**value**	**strand**	**id**	**edges^2^**

**Type of column:**	**N**	**N**	**C**	**C**	**C**	**N**	**N**	**C**
**Track type**								
Points (P)	?	!	✓	.	.	?	?	.
Segments (S)	?	!	✓	✓	.	?	?	.
Genome Partition (GP)	?	!	.	✓	.	?	?	.
Valued Points (VP)	?	!	✓	.	✓	?	?	.
Valued Segments (VS)	?	!	✓	✓	✓	?	?	.
Step Function (SF)	?	!	.	✓	✓	?	?	.
Function (F)	?	!	.	.	✓	?	?	.
Linked Points (LP)	?	!	✓	.	.	?	✓	✓
Linked Segments (LS)	?	!	✓	✓	.	?	✓	✓
Linked Genome Partition (LGP)	?	!	.	✓	.	?	✓	✓
Linked Valued Points (LVP)	?	!	✓	.	✓	?	✓	✓
Linked Valued Segments (LVS)	?	!	✓	✓	✓	?	✓	✓
Linked Step Function (LSF)	?	!	.	✓	✓	?	✓	✓
Linked Function (LF)	?	!	.	.	✓	?	✓	✓
Linked Base Pairs (LBP)	?	!	.	.	✓	?	✓	✓

C Core reserved column (defines track type)

N Non-core reserved column (reserved, but does not define track type)

✓ Column is mandatory

? Column is optional

. Column is not allowed

! Property must be present, either as a column or in a bounding region specification

^1 ^The length is the difference between the end and the start position, or, if the start column is not present, the difference between the current end position and the previous.

^2 ^The non-core reserved column *id *is required when the edges column is present.

• Gaps are implicitly represented by the *start *column, *i.e*. it holds the start coordinate of a track element and thus marks the end of any preceding gap.

• For *sparse *track types, *i.e*. track types with gaps, length is implicitly represented by the difference between *start *and *end *columns. For *dense *track types (without gaps), there is no *start *column. The length is then the difference between the previous *end *position and the current. Deriving length from the *end *position, rather than the *start *position, is preferable, as a parser in the opposite case would have to read the subsequent line before concluding on the length of the current track element. The existence of the *end *column thus corresponds directly to the track elements having the length property.

• Although several columns in a data set may contain values of potential interest, one of these columns will typically provide a main value used in processing or analysis according to a given purpose. This focus is specified by the *value *column.

• The *edges *column contains, for each track element, a comma-separated list of id's of other track elements which are interconnected with the element in question, in addition to values associated to the edges, *e.g*. weights or edge types

• A GTrack file may contain several columns containing values or edges. Users may then switch between them by simply editing the column specification line.

**Figure 4:**
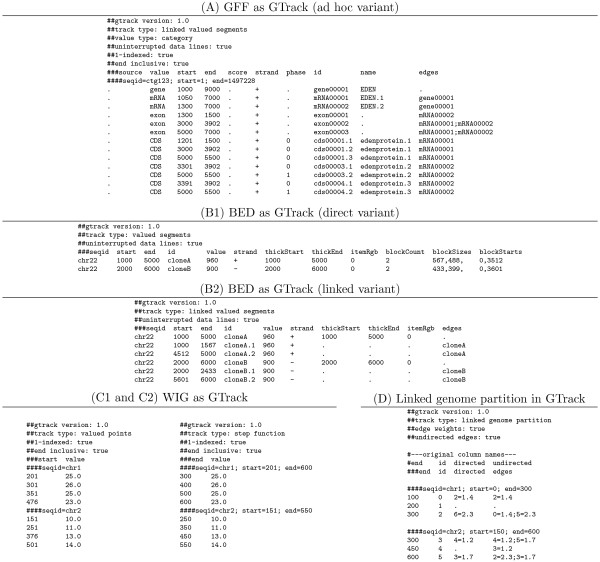
**GTrack example files**. A) GTrack version of the GFF file in Figure 3A. GTrack conversions of GFF vary according to the set of attributes present in the GFF file. The column selected as the main value may also be changed. B1 and B2) Two possible GTrack conversions of the BED file in Figure 3B. In the direct variant (B1) only a "track type" header line and a column specification line are added. The exon positioning will in this case not be understood by a general GTrack parser. The linked variant (B2) expands the exons into subsegments that links to their parent gene segment. C1 and C2) GTrack conversions of the WIG files in Figure 3C. The *variableStep *file has sparse track elements covering single base pairs, with associated values. The track is thus of type *valued points*. The *fixedStep *file contains dense data, with the same values for a series of consecutive base pairs. The track type is thus of type *step function*. Note that in the last example, the end values are used for positioning. D) Example GTrack file of type *linked genome partition*. Here two graphs are defined, one directed and one undirected. To change the active graph, the *edges *column in the column specification line needs to be changed, in addition to the "undirected edges" header line. The example GTrack files are available at [20]. BioXSD 1.1 versions of the examples are available as follows: A [21], B1 & B2 [22], C1 [23], C2 [24], and D [25].

The *edges *column requires that the non-core reserved column *id *is present, containing a unique identifier for each track element. Three other non-core columns are specified in the GTrack format: *genome*, *seqid *and *strand *(see Table 3). The titles of the eight reserved columns are reserved words in the column specification line. They may appear in any order, and any number of additional columns may be specified. Figure 4 shows six example GTrack files, five of which are conversions of the example files in Figure 3. The example files illustrate the variation stemming from the different column specification lines (starting with the characters '###').

When creating the GTrack format, we have emphasized simplicity, both for creation, manual reading and automated parsing of the format. We have identified three principles towards simplicity: independence of data lines, overview of structural characteristics and equally sized lines.

The principle of independent data lines states that it should be possible to interpret each data line in a tabular format independently of its location in the file. This is a principle followed in many common formats, *e.g*. GFF [2] or BED [4]. Following this principle gives several advantages. First, when creating or manipulating a file, keeping data lines independent allows the filtering and sorting of data lines while still keeping all the relevant information. Second, keeping a track element on a single line makes it easier to read for the human eye. Third, independent data lines reduce the need of automatic parsers to hold state information. The GTrack format follows the principle of independent data lines with two exceptions. First, data lines of dense track types are dependent on their positions in the file. Second, the GTrack format allows (and, in the case of dense track types, requires) the specification of bounding regions around each block of values. A bounding region specification line defines the domain of the following track elements, *i.e*. the region where we have information about the features modeled by the track elements. It is recommended that tracks mask out regions of a genome where nothing is known (such as centromeres or assembly gaps) using bounding regions, rather than just omitting track elements or specifying 0-values, as the difference is important for many analyses. Bounding regions unfortunately require parsers to store state information. See Figure 4A, 4C1, 4C2 and 4D for examples of bounding region specification lines (starting with the characters '####').

The principle of including an overview of structural characteristics means that a track file should start with a set of configurable options that describe the structure of the data lines, in an easily readable manner. Note that many of these characteristics will, by nature, include redundant information, *i.e*. that could have been collected from the data lines themselves. There are several reasons for explicitly stating such characteristics. First, it gives the human reader a simple overview of the type of data stored in the file, without having to scrutinize the actual data. Second, it allows the creator of a track to validate that the file is structured in the way intended (for this purpose, we also provide a web-based validator tool [20]). Third, inclusion of structural characteristics allows parsers to be restrictive on which kind of structures to support. A quick script can then, for instance, read the header and check whether the track type is *segments *with no overlapping elements, failing explicitly if the header does not match this requirement. The script can then assume that the remaining file follows the asserted structure, safely ignoring the non-relevant generality of the GTrack specification. In the GTrack format, the structural characteristics are specified in header lines, starting with the characters '##'. Table 4 contains an overview of all GTrack header variables. Note that header lines are optional when their values are equal to the default values. We also provide the "Expand GTrack headers" tool, which generates a GTrack file with full headers based on a supplied, incomplete GTrack file, further simplifying the process of generating header lines.

**Table 4:** Overview of the header variables of the GTrack format

Header variable	Description	Default value
GTrack version	Version of the GTrack specification used	1.0
Track type	Track type of the GTrack file	segments
Value type	The kind of content accepted in the value column	number
Value dimension	The dimension of the content in the value column	scalar
Undirected edges	Whether all edges are undirected	false
Edge weights	Whether the edges have weights	false
Edge weight type	The kind of content accepted as edge weights	number
Edge weight dimension	The dimension of the edge weights	scalar
Uninterrupted data lines	Whether it is guaranteed that the data lines are not interrupted by bounding region specification lines or comments	false
Sorted elements	Whether it is guaranteed that all bounding regions and track elements come in sorted order	false
No overlapping elements	Whether it is guaranteed that no two track elements overlap	false
Circular elements	Whether any track elements or bounding regions cross the coordinate borders of a circular sequence	false
1-indexed	Whether the coordinates start at 1 (0 if false)	false
End inclusive	Whether the coordinates specified in the end column is included in intervals	false
*Value column	The name of the column to be used for as the 'value' column	value
*Edges column	The name of the column to be used for as the 'edges' column	edges
*Fixed length	Fixed length of all track elements	1
*Fixed gap size	Fixed-size gaps between all neighboring track elements	0
*Fixed-size data lines	Whether each data line has an exact size in terms of number of characters	false
*Data line size	The size of each data line in terms of number of characters	1
*GTrack subtype	The name of the subtype of the GTrack format specification used for the file	(empty string)
*Subtype version	The version of the GTrack subtype	1.0
*Subtype URL	URL to a GTrack file used as a specification/model for the GTrack subtype	(empty string)
*Subtype adherence	Regulates the way a GTrack file may override the subtype specification	free

All header variables not specified in a GTrack file retains their default values.

* Defined in the extended part of the GTrack specification. See the GTrack specification (Additional file 1) for more details.

The principle of equally sized lines states that all data lines contain the same number of columns, *i.e*. that all attributes have a value. Columns that do not contain information are marked with a period character. There are several advantages for this solution compared to the solution used in the GFF format, where the last column may contain a list of attributes in the format tag = value, allowing the attribute list to differ for each line. First, having equal size columns allows validation that all data lines are complete, or at least that the creator of the track has considered all attributes for all track elements. With a variable size attribute column, there is no way to check that all attributes have been considered. Second, parsing attribute lists as in the GFF format is more cumbersome, as the parser will not in advance know which attributes may appear in the file. Third, not having to repeat attribute names for all lines saves some space. Fourth, and most importantly, having the same number of columns in each data line keeps the interface of the format coherently organized, with attributes as columns and track elements as rows. As the GTrack format supports custom columns, it can completely replace the attribute solution of the GFF format.

**Figure 5:**
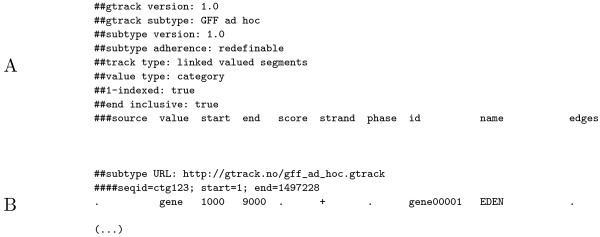
**GTrack subtype example**. A) An *ad hoc *GTrack suptype specification based on the example GTrack file in Figure 4A, which is a conversion from the GFF file in Figure 3A. This and other GTrack subtypes are available from the GTrack website [20]. B) A minimal GTrack header, parsable by fully compliant GTrack parsers. Note that the "Expand GTrack headers" tool, available from the GTrack website [20], can be used to expand headers of GTrack files using subtypes, in order for such files to be used in simpler parsers that do not support the subtype functionality.

In addition to simplicity, the GTrack format aims at being highly extensible and inter-operable. First, the ability to define columns in any order and number, provides ample options for extensibility, in addition to simplifying conversion. In many cases, converting another tabular format to GTrack is as simple as adding a column specification line. Note that basic, three-column BED files are directly compatible with the GTrack format, without the need for any modifications. Also, both 0- and 1-based indexing, in addition to the *end *position being inclusive or exclusive, are included in the GTrack specification, further simplifying conversion. Second, GTrack includes a strategy for making structured extensions of the format, namely the specification of subtypes. Four subtype header lines are available (see Table 4), specifying the name and version of a subtype, the URL of the subtype specification, and the strictness of adherence required by the subtype. The idea is that research communities can define their own tabular formats, making use of a subset of the GTrack specification. Such formats could for instance be replacements of existing formats, or formats that are honed to specific technologies or tools. The header variable "subtype URL" points to a GTrack file that can be used as model for the subtype, and is intended to be read by automatic parsers. Figure 5A shows an example of such a subtype specification file, based on the example GTrack file in Figure 4A. Specifying subtype models allows the reduction of a complete GTrack header down to a minimum of one line, as shown in Figure 5B. It is our belief that allowing extensions of the GTrack format via subtypes caters for a range of future extensions, while ensuring backward compatibility. Subtypes can be defined in a range of settings, from project specific, *ad hoc *solutions, to the specification of generic formats. Further examples of GTrack subtypes are described in the GTrack specification (Additional file 1). A set of standard GTrack subtypes are available online [20] (including subtypes corresponding to the example files in Figure 4).

### BioXSD 1.1: Enhanced and optimized XML format

BioXSD has been developed as a universal XML format for the basic types of bioinformatics data that is in particular suitable to be used with Web services [16]. It models common types of data for which a specialized XML Schema (XSD) has not been widely adopted: biomolecular sequences, alignments, sequence feature records, and references to ontologies and data resources. The BioXSD schema defines formats of data but not formats of particular XML documents, by defining XSD types but no global XML elements. BioXSD types can thus be used according to applications' needs in applications' own XSDs such as those in WSDL files of Web services.

BioXSD 1.0 type AnnotatedSequence can represent annotations of a biomolecular sequence or genome with any types of positioned or non-positioned features, which can be combined in one record. Although the textual serialization of XML is in general more verbose than a tabular format, already the BioXSD 1.0 has included a number of optimizations compared to traditional feature formats like GFF or BED, thanks to the tree-like structure of XML. These have been mainly:

• not repeating the reference to a sequence in every *feature occurrence*

• not repeating the *type of feature *in every feature occurrence

• representing multi-segment and multi-point feature occurrences in one feature-occurrence element

The goal of BioXSD version 1.1 has been to further improve the expressiveness of the BioXSD formats and at the same time focus on optimizations of the data size. The successor of BioXSD 1.0 AnnotatedSequence is BioXSD 1.1 type FeatureRecord. BioXSD 1.1 in general allows more types of sequence positions, distinguishing them in the same way as the tabular GTrack format. Sparse positions are *segments*, *points *(actual points or insertions), and outer positions. Dense positions have been added: dense points (*function*) marked-up by < nextPoint/> empty elements; and dense *partition *or *step function *marked by < nextPartition max="..."/> elements including the border position where each interval ends. However in contrast to GTrack, the different types of positions can still be freely combined within a FeatureRecord. The representation of all types of sequence positions have been refactored, simplified, and optimized. Another crucial set of optimizations allows specification of the ontologies, databases, and computational tools of interest in a condensed way for a list of feature annotations, so that they do not have to be repeated. Detailed contents of the BioXSD feature record are listed in Table 5. Examples of data represented in BioXSD 1.1 format are available at [21-25].

**Table 5:** The allowed content of a BioXSD FeatureRecord

	Notes	May further contain
**BioXSD description of feature type**		
Name	^1 ^	
Ontology concepts	^1 ^	
Synonyms		
Textual note		
References	to database entries, databases, ontology concepts, other feature types	type of relationship with the referenced object^2^
More specific type of feature		name and/or concepts, synonyms, database entries
More generic class of feature types		name and/or concepts
		

**BioXSD feature occurrence**		
Position	*segments*, *points*^3^, positions outside of the actual sequence or feature occurrence^4^, dense points* (*function*) and dense partition* or step function* ^5^	strand, certainty
Scores (*values*)	double-precision signed floats (8 bytes), or any well-formatted strings*	unit, index, type of score^2^, note, position, provenance metadata
Evidence		references to databases, tools, and citations; scores, verdict, reliability, provenance metadata
Name		
Note		
Alignments		alignment- and aligned sequence-specific scores, gaps, frameshifts, directions, note, provenance metadata
Sequence variation		variants, canonical variant, scores, position
Frame		
CDS phase		
References	to ontology concepts, database entries, other feature occurences (*interconnections*)	type of relationship with the referenced object^2^; scores of the relationship (*weights *of *edges*)*

^1 ^At least one of these two is mandatory.

^2 ^By any ontology concept, referred to by a concept URI, identifier, or term; or by a custom term if no ontology concept is available.

^3 ^Points are bases/residues or insertions between them.

^4 ^For example if annotating the position of a regulatory element of a coding sequence, or relations between genes or protein domains.

^5 ^Positions can form multi-segment subsequences, multi-point tuples, and can be combined within feature occurrences according to users' needs. The positions are always 1-based. The feature occurence may apply to the whole sequence (being a non-positioned sequence property).

* Added in BioXSD version 1.1.

There is one slight difference in how the GTrack and BioXSD deal with *focus *of feature records. GTrack defines one operational focus of a concrete dataset. That is the reason why it allows to specify only one type of track locations and only one *value *column and one *edges *column at a time, although other values and edges may still be "hidden" in out-of-focus columns. BioXSD on the other hand allows combining features, types of track positions, values, and interconnections freely without any operational focus. Thus, if a tool consuming BioXSD feature data demands it, a particular operational focus of the data must be supplied by the user.

Compared to other generic sequence-feature formats, BioXSD allows defining complex, structured meanings of annotations, as well as complex feature data and metadata, or relations. This would not be conveniently possible in a tabular format and takes advantage of the XML. BioXSD types can freely be combined and included within documents, files, or applications' inputs and outputs. They can easily be combined with other XML formats defined in other XSDs, can be extended just like classes in an object-oriented programming language, or further restricted using built-in XSD mechanisms. BioXSD can be validated and parsed by ordinary XML/XSD-handling frameworks.

It has, however, been problematic to use XML formats for highly voluminous data such as whole-genome annotations. The textual serialization of XML is more verbose compared to a textual tabular format, and even more compared to a bespoke binary format. Many basic XML-handling tools have high runtime demands for computer memory, making parsing of huge XML documents impossible. All these problems are hopefully going to be solved thanks to the recent and long-expected Efficient XML Interchange (EXI) standard by the World Wide Web Consortium [17], together with its growing family of supporting libraries, and tools for streamed XSLT transformations and random-access XPath and XQuery queries. EXI defines the way any XML data or document should be serialized in a standard binary format that will be many times smaller and at the same time faster to access than the textual XML. There is no need to develop one's own bespoke binary encodings and parsers when using EXI, and the data can be programmatically handled transparently, with the same look and feel as the ordinary XML.

### Availability of specifications and supporting tools

The BioXSD 1.1 XML Schema is available at [26]. BioXSD data can be validated by all the main XML validation tools, and consumed and produced programmatically by the bulk of the common XML/XSD-handling libraries. Further information and documentation are available at [27].

A complete specification of the GTrack format version 1.0 is attached as Additional file 1 and is also available from the GTrack website [20]. The website also contains supporting tools for the GTrack format, connected to the Genomic HyperBrowser [10,28]. Table 6 contains an overview of all GTrack-related tools available as webtools.

**Table 6:** Overview of the webtools available from the GTrack website [20]

GTrack supporting tools	Description
Show GTrack specification	Displays a HTML version of the GTrack specification
Validate GTrack file	Checks whether a GTrack file complies with the specification
Convert tabular file to GTrack	Converts any tabular file to GTrack
Convert file to/from GTrack	Converts to and from common tabular formats (GFF, BED, WIG, bedGraph)
Expand GTrack headers	Expands partially completed GTrack headers based on data contents
Standardize GTrack file	Converts a GTrack file to track type "linked valued segments" using the default indexing scheme
Sort GTrack file	Sorts a GTrack file (including bounding regions)
Complement GTrack columns	Complements the columns of a GTrack file based on another GTrack file

All tools are implemented as part of the Genomic HyperBrowser [10,28] and available under the GPL license, version 3 [34].

The GTrack format is maintained by Sveinung Gundersen and the BioXSD format is maintained by Matúš Kalaš. Both formats are licensed under the Creative Commons Attribution-NoDerivs 3.0 Unported License [29].

The Genomic HyperBrowser [10,28] is built on top of the Galaxy framework [30,31] and provides a large set of statistical investigations tailored for the specific track types of supplied tracks. In order for such analyses to be efficient, the system uses a binary storage scheme internally. In this scheme, the core informational columns are stored as C vectors directly written to disk. The vector files are then accessed using the NumPy package [32] for Python [33], allowing very efficient vector computations. A linear index of the files is built in order to allow random access to the data. This binary representation is stored in parallel to the files in their original format, and updated automatically as the original files are updated. The implementation is open source and available as part of the HyperBrowser code base under the GPL license, version 3 [34]. As an alternative, the recently published Tabix tool [35] provides fast access to tabular data in compressed form, and works with GTrack files of types Points and Segments, and their derivatives.

## Conclusions

By systematic analysis of informational properties of genomic tracks, we delineated fifteen distinct types of tracks. These track types shed light on the variability of track representations, suggesting that the differences between formats is not only due to preferences and conventions, but also to fundamental differences in the information inherent in different tracks. Furthermore, discerning the informational properties of a track allows the nature of the track to be precisely conveyed, as well as clarifying what represents meaningful analyses on a given track.

The identification of core informational properties of tracks, as well as a broad survey of various practicalities concerning existing formats, created a basis for the specification of a new format for genomic data: the GTrack format. By allowing precise interpretation, simple parsing, as well as relatively straightforward conversion to several existing formats, we believe that the introduction of this "yet another format" will actually help streamline data representation in the field. Finally, by coordinating the GTrack format with an enhanced and optimized version 1.1 of the BioXSD format, this also aids in unifying tabular and XML-based track representation, while keeping the specific advantages of the two.

## Abbreviations

BAM: Binary Alignment/Map format; BED: Browser Extensible Data format; ChIP-seq: Chromatin Immunoprecipitation sequencing; EXI: Efficient XML Interchange; F: function; GFF: General Feature Format; GTF: Gene Transfer Format; GVF: Genome Variation Format; GP: genome partition; P: points; LBP: linked base pairs; LF: linked function; LGP: linked genome partition; LP: linked points; LS: linked segments; LSF: linked step function; LVP: linked valued points; LVS: linked valued segments; S: segments; SAM, Sequence Alignment/Map format; SF: step function; SNP: single nucleotide polymorphisms; URI: Uniform resource identifier; URL: Uniform resource locator; VP: valued points; VS: valued segments; WIG: Wiggle format; WSDL: Web Service Definition Language; WYSIWYG: what you see is what you get; XML: Extensible Markup Language; XSD: XML Schema Definition.

## Authors' contributions

SG, AF, EH and GKS conceived and developed the ideas on track type distinctions. SG, MK, OA and GKS developed the GTrack specification. SG and GKS wrote the main parts of the paper. MK wrote the parts on XML-based track representation and developed BioXSD 1.1. SG and GKS were involved with the development of GTrack-related tools. All authors read and approved the final manuscript.

## Supplementary Material

Additional file 1 **GTrack specification**. Specification document of GTrack 1.0. Click here for file

## References

[B1] Lieberman-Aiden E van Berkum NL Williams L Imakaev M Ragoczy T Telling A Amit I Lajoie BR Sabo PJ Dorschner MO Sandstrom R Bernstein B Bender MA Groudine M Gnirke A Stamatoyannopoulos J Mirny LA Lander ES Dekker J Comprehensive mapping of long-range interactions reveals folding principles of the human genome Science 2009 326 5950 289 293 10.1126/science.1181369 19815776 PMC2858594

[B2] Generic Feature Format version 3 http://www.sequenceontology.org/gff3.shtml

[B3] Kent WJ Sugnet CW Furey TS Roskin KM Pringle TH Zahler AM Haussler D The human genome browser at UCSC Genome Res 2002 12 6 996 1006 12045153 10.1101/gr.229102 PMC186604

[B4] UCSC genome browser data formats http://genome.ucsc.edu/FAQ/FAQformat.html

[B5] Definition of Gene Transfer Format http://mblab.wustl.edu/GTF22.html

[B6] Reese MG Moore B Batchelor C Salas F Cunningham F Marth GT Stein L Flicek P Yandell M Eilbeck K A standard variation file format for human genome sequences Genome Biol 2010 11 8 R88 10.1186/gb-2010-11-8-r88 20796305 PMC2945790

[B7] Liu F Tostesen E Sundet JK Jenssen TK Bock C Jerstad GI Thilly WG Hovig E The human genomic melting map PLoS Comput Biol 2007 3 5 10.1371/journal.pcbi.0030093 PMC1868775 17511513

[B8] Definition of Wiggle Track Format http://genome.ucsc.edu/goldenPath/help/wiggle.html

[B9] The Sequence Ontology http://www.sequenceontology.org

[B10] Sandve GK Gundersen S Rydbeck H Glad IK Holden L Holden M Liestol K Clancy T Ferkingstad E Johansen M Nygaard V Tostesen E Frigessi A Hovig E The Genomic HyperBrowser: inferential genomics at the sequence level Genome Biol 2010 11 12 R121 10.1186/gb-2010-11-12-r121 21182759 PMC3046481

[B11] Li H Handsaker B Wysoker A Fennell T Ruan J Homer N Marth G Abecasis G Durbin R The Sequence Alignment/Map format and SAMtools Bioinformatics 2009 25 16 2078 2079 10.1093/bioinformatics/btp352 19505943 PMC2723002

[B12] Dowell RD Jokerst RM Day A Eddy SR Stein L The distributed annotation system BMC Bioinformatics 2001 2 7 10.1186/1471-2105-2-7 11667947 PMC58584

[B13] Web services provided by the Center for Biological Sequence analysis (CBS), Technical University of Denmark http://www.cbs.dtu.dk/ws/

[B14] UniProt C The Universal Protein Resource (UniProt) in 2010 Nucleic Acids Res 2010 38 Database Issue D142 8 10.1093/nar/gkp846 PMC2808944 19843607

[B15] Gould CM Diella F Via A Puntervoll P Gemund C Chabanis-Davidson S Michael S Sayadi A Bryne JC Chica C Seiler M Davey NE Haslam N Weatheritt RJ Budd A Hughes T Pas J Rychlewski L Trave G Aasland R Helmer-Citterich M Linding R Gibson TJ ELM: the status of the 2010 eukaryotic linear motif resource Nucleic Acids Res 2010 38 Database Issue D167 80 10.1093/nar/gkp1016 PMC2808914 19920119

[B16] Kalas M Puntervoll P Joseph A Bartaseviciute E Topfer A Venkataraman P Pettifer S Bryne JC Ison J Blanchet C Rapacki K Jonassen I BioXSD: the common data-exchange format for everyday bioinformatics web services Bioinformatics 2010 26 18 i540 6 10.1093/bioinformatics/btq391 20823319 PMC2935419

[B17] Efficient XML Interchange (EXI) Format 1.0 http://www.w3.org/TR/2011/REC-exi-20110310

[B18] Kent WJ Zweig AS Barber G Hinrichs AS Karolchik D BigWig and BigBed: enabling browsing of large distributed datasets Bioinformatics 2010 26 17 2204 2207 10.1093/bioinformatics/btq351 20639541 PMC2922891

[B19] Nix DA Courdy SJ Boucher KM Empirical methods for controlling false positives and estimating confidence in ChIP-Seq peaks BMC Bioinformatics 2008 9 523 10.1186/1471-2105-9-523 19061503 PMC2628906

[B20] GTrack http://www.gtrack.no

[B21] BioXSD example 1 http://bioxsd.org/trackExample1.xml

[B22] BioXSD example 2 http://bioxsd.org/trackExample2.xml

[B23] BioXSD example 3 http://bioxsd.org/trackExample3.xml

[B24] BioXSD example 4 http://bioxsd.org/trackExample4.xml

[B25] BioXSD example 5 http://bioxsd.org/trackExample5.xml

[B26] Definition of BioXSD version 1.1 http://bioxsd.org/BioXSD-1.1.xsd

[B27] BioXSD.org http://bioxsd.org

[B28] The Genomic HyperBrowser http://hyperbrowser.uio.no

[B29] Creative Commons Attribution-NoDerivs 3.0 Unported License (CC BY-ND 3.0) http://creativecommons.org/licenses/by-nd/3.0/

[B30] Goecks J Nekrutenko A Taylor J Galaxy: a comprehensive approach for supporting accessible, reproducible, and transparent computational research in the life sciences Genome Biol 2010 11 8 R86 10.1186/gb-2010-11-8-r86 20738864 PMC2945788

[B31] Blankenberg D Von Kuster G Coraor N Ananda G Lazarus R Mangan M Nekrutenko A Taylor J Galaxy: a web-based genome analysis tool for experimentalists Curr Protoc Mol Biol 2010 19 21 Unit 19.10.1 10.1002/0471142727.mb1910s89 PMC4264107 20069535

[B32] Oliphant T Guide to NumPy 2006 Trelgol Trelgol Publishing

[B33] The Python Language Reference http://docs.python.org/release/2.7.2/reference/index.html

[B34] GNU General Public License, version 3 http://www.gnu.org/copyleft/gpl.html

[B35] Li H Tabix: fast retrieval of sequence features from generic TAB-delimited files Bioinformatics 2011 27 5 718 719 10.1093/bioinformatics/btq671 21208982 PMC3042176

[B36] Affymetrix CNT File Format http://goldenhelix.com/SNP_Variation/Manual/svs7/affymetrix_cnt_file_format.html

[B37] VCF (Variant Call Format) version 4.1 http://www.1000genomes.org/wiki/Analysis/Variant%20Call%20Format/vcf-variant-call-format-version-41

[B38] The SAM Format Specification (v1.4-r985) http://samtools.sourceforge.net/SAM1.pdf

[B39] BioHDF http://www.hdfgroup.org/projects/biohdf/

[B40] FASTA http://www.ncbi.nlm.nih.gov/BLAST/blastcgihelp.shtml

[B41] Hoffman MM Buske OJ Noble WS The Genomedata format for storing large-scale functional genomics data Bioinformatics 2010 26 11 1458 1459 10.1093/bioinformatics/btq164 20435580 PMC2872006

